# Comparison between sustained low-efficiencydialysis (SLED) and continuous renal replacement therapy (CRRT) in patients of septic shock: a randomized controlled trial

**DOI:** 10.1186/2197-425X-3-S1-A55

**Published:** 2015-10-01

**Authors:** SB Mishra, A Azim, AK Baronia, RK Singh, M Gurjar, B Poddar

**Affiliations:** Sanjay Gandhi Post Graduate Institute of Medical Sciences, Critical Care Medicine, Lucknow, India

## Introduction

Acute Kidney Injury is common in patients of septic shock. There is sparse data comparing SLED and CRRT in septic shock patients.

## Objectives

Compare the hemodynamic stability and outcome of SLED vs. CRRT in septic shock patients.

## Methods

Prospective randomized study in a 12 bedded medical ICU. After clearance from institutes ethics committee and taking informed consent from the relatives 40 adult patients in septic shock who were to undergo dialysis for Acute Kidney Injury were included in the study. They were randomly assigned to SLED or CRRT group. They underwent the same modality of dialysis until they were hemodynamically stable. Study was done with an Intention to treat. Hemodynamic instability was defined as an increase in vasopressor dependency > 20 mmHg-1 from the pre dialysis value. Vasopressor index was calculated by the formula (dopamine dose×1) + (dobutamine dose×1) + (adrenaline dose×100) + (noradrenaline dose×100) + (vasopressin dose×10).The doses were in microgram/kg/min for all vasopressors except vasopressin which is in Units/hour. The Vasopressor dependency was calculated by the formula (Vasopressor index/MAP) X 100. The worst value of vasopressor dependency during the dialysis session was taken into consideration. The primary objective was 28 day mortality. The secondary objective was hemodynamic stability during the dialysis sessions. The Statistical analysis was done by SPSS 17. Mann Whitney test was done for continuous data and Fishers Exact test was done for proportions. Survival analysis was done by Kaplan Mayer method with log rank test.

## Results

The study was conducted from June 2014 to January 2015. The two groups were comparable in terms of age, sex, APACHE II & SOFA (p > 0.05).(Table 1) Pre dialysis vasopressor dose, BUN, Creatinine, Vasopressor index, Vasopressor dependency, & base deficit were also comparable (p > 0.05). (Table 2) The 28 day mortality rate was 75% (15/20) in CRRT group and 65% (13/20) in SLED group. The log rank estimate was 0.64 for mortality. The hemodynamic instability as defined by vasopressor dependency was 54% (13/24) in CRRT group while in SLED group it was 60% (27/45) (p= 0.50).(Table 3)

**Table 1:** Demographics.

	SLED (n = 20) Median(IQR)	CRRT (n = 20) Median (IQR)	p value
Age (years)	54 (29 - 60)	47 (24 - 62)	0.85
Sex (male)-n (%)	15 (75%)	12 (60%)	0.50
APACHE II	25 (20 - 28)	28 (22 - 29)	0.35
SOFA	13 (10 - 15)	14 (12 - 18)	0.09

**Table 2:** Characteristics at the time of Randomization.

	SLED (n = 20) Median(IQR)	CRRT (n = 20) Median (IQR)	p value
Creatinine (mg/dl)	2.5 (1.5 - 4.1)	3 (1.4 - 4.5)	0.90
BUN (mg/dl)	71 (53 - 100)	46 (37 - 125)	0.54
MAP (mmHg)	81 (70 - 87)	78 (73-99)	0.45
Dose of Noradrenaline	0.3 (0.21-0.61)	0.35 (0.3 - 0.75)	0.56
Bicarbonate (mmol/L)	18 (14 - 23)	16 (14 - 18)	0.68
Base deficit	8 (3 - 11)	12 (5-13)	0.10
Vasopressor Index	50 (43 - 84)	62 (52 -104)	0.14
Vasopressor Dependency	64 (45 - 105)	75 (55 - 98)	0.42

**Table 3:** Outcome.

	SLED (n = 20) Median(IQR)	CRRT (n = 20) Median (IQR)	p value
Mortality (Day 28)	13/20 (65%)	15/20 (75%)	0.64
Length of stay (in ICU)	13.5 (5 - 30)	12 (6 - 25)	0.73
Hemodynamic unstable sessions	27/45 (60%)	13/24 (54%)	0.50

## Conclusions

SLED is comparable to CRRT in septic shock patients and can be used as an alternative modality of RRT. Larger RCTs are needed to confirm the above finding.
